# The Critical Role of Hypoxic Microenvironment and Epigenetic Deregulation in Esophageal Cancer Radioresistance

**DOI:** 10.3390/genes10110927

**Published:** 2019-11-14

**Authors:** Catarina Macedo-Silva, Vera Miranda-Gonçalves, Rui Henrique, Carmen Jerónimo, Isabel Bravo

**Affiliations:** 1 Cancer Biology & Epigenetics Group—Research Center at Portuguese Oncology Institute of Porto (CI-IPOP), 4200-072 Porto, Portugal; ana.catarina.macedo.silva@ipoporto.min-saude.pt (C.M.-S.); vera.miranda.goncalves@ipoporto.min-saude.pt (V.M.-G.); henrique@ipoporto.min-saude.pt (R.H.); 2 Department of Pathology at Portuguese Oncology Institute of Porto (CI-IPOP), Portugal, 4200-072 Porto, Portugal; 3 Department of Pathology and Molecular Immunology at Institute of Biomedical Sciences Abel Salazar—University of Porto (ICBAS-UP), 4050-313 Porto, Portugal; 4 Medical Physics, Radiobiology and Radiation Protection Group—Research Center at Portuguese Oncology Institute of Porto (CI-IPOP), 4200-072 Porto, Portugal

**Keywords:** Epigenetic, esophageal cancer, hypoxia, microenvironment, radiobiology, radioresistance

## Abstract

Esophageal cancer (EC) is the seventh most common cancer worldwide and the sixth leading cause of death, according to Globocan 2018. Despite efforts made for therapeutic advances, EC remains highly lethal, portending a five-year overall survival of just 15–20%. Hence, the discovery of new molecular targets that might improve therapeutic efficacy is urgently needed. Due to high proliferative rates and also the limited oxygen and nutrient diffusion in tumors, the development of hypoxic regions and consequent activation of hypoxia-inducible factors (HIFs) are a common characteristic of solid tumors, including EC. Accordingly, HIF-1α, involved in cell cycle deregulation, apoptosis, angiogenesis induction and proliferation in cancer, constitutes a predictive marker of resistance to radiotherapy (RT). Deregulation of epigenetic mechanisms, including aberrant DNA methylation and histone modifications, have emerged as critical factors in cancer development and progression. Recently, interactions between epigenetic enzymes and HIF-1α transcription factors have been reported. Thus, further insight into hypoxia-induced epigenetic alterations in EC may allow the identification of novel therapeutic targets and predictive biomarkers, impacting on patient survival and quality of life.

## 1. Introduction

Esophageal cancer (EC) incidence has increased in recent decades, representing the seventh most common cancer worldwide and the sixth cause of cancer-related death, according to Globocan 2018 [1], with an overall five-year survival ranging from 15–20% [2,3]. There are two major EC subtypes—Adenocarcinoma (EAC) and squamous cell carcinoma (ESCC)—Which disclose unlike geographical distributions as a consequence of differentially associated risk factors and ethnicity [4,5,6]. Indeed, tobacco, alcohol consumption and dietary habits constitute the main risk factors for ESCC, whereas, Barrett’s esophagus (BE), gastroesophageal reflux disease (GERD) and obesity are associated with EAC [7].

Despite major advances in patients’ management and therapeutic strategies, including multimodality neoadjuvant approaches, the prognosis of advanced stage EC remains poor [8,9]. Currently, localized tumors are primarily treated with radical surgery, with an expected five-year survival rate of 10–40% [8]. Nonetheless, most ECs are diagnosed at advanced stages, in which primary surgery is not an effective therapy [9], entailing the need for multimodal treatments. In this setting, neoadjuvant chemoradiotherapy (CRT) achieves the highest complete pathological response rates, significantly improving EC patients’ overall outcomes [8,9,10]. Moreover, a synergistic effect is achieved, combining radio- and chemotherapy with surgical resection, i.e., trimodal therapy [8].

Discovery of cancer-specific molecular targets has been a major focus of research to improve cancer survival rates [11]. Taking into account tumor biology and microenvironment heterogeneity, tumors respond differently to radiotherapy (RT), presenting several forms of therapy resistance [12]. Since RT acts through a fundamentally biological, rather than physical, mechanism both the understanding of how radiation influences tumor cells’ behavior, and its surrounding microenvironment is required to maximize therapeutic efficacy.

A common feature of solid tumors, including EC, is the presence of hypoxic regions, and its extent has been previously correlated with poor prognosis [13].

It is well known that molecular oxygen local availability permanently fixes radiation induces DNA lesions produced by free radicals, leading to unrepairable damage, and thus, enhancing RT efficacy. Moreover, one of the cancer cells’ goal consists in the maintenance of a pool of reductive molecules that protect against redox stress [14]. RT causes a redox imbalance in favor of excessive oxidation [14], and must overcome the redox defenses of tumor cells in order to be effective. In this context, hypoxia is highly detrimental as it interferes with the fixation of DNA damage causing resistance to RT.

A more detailed characterization of the molecular alterations associated with hypoxia could, not only, serve as a surrogate marker of this phenomenon, but also as relevant therapeutic targets, eventually impacting in EC response to CRT. Furthermore, epigenetic alterations are a recognized cancer hallmark owing to its role in altered chromatin organization and consequent transcriptional gene deregulation [15,16]. Moreover, it has been suggested that hypoxic tumor microenvironment might unleash several epigenetic changes resulting in increased tumor aggressiveness and RT resistance [17]. In radioresistant tumor cells, a phenotypic switch occurs during the course of radiation delivery, associated with both genetic and epigenetic changes [18]. Nonetheless, there is only limited evidence regarding the usefulness of epigenetic alterations as a potential predictor of response to radiotherapy [19]. Thus, the aim of this review is to summarize published evidence on the role of hypoxia-induced epigenetic alterations which might impact on RT resistance in EC.

## 2. Biological Basis of Cancer Radiation Therapy

RT plays an important role in improving the survival of EC patients who are not eligible for surgery [9]. The standard dose recommended by European and North American guidelines is 50.4 Gy as total dose [20], in 28 fractions of 1.8 Gy/day. Regarding Asian countries, the standard dose recommended is a total treatment of 60 Gy [20]. Although RT efficacy has developed considerably in recent years, due to advances in precision techniques, such as intensity-modulated RT (IMRT) or volumetric-modulated arc therapy (VMAT), inter-individual differences are observed, and significant response is not achieved in some patient subgroups. Cancer cells exposed to radiation, depending on the surrounding tumor microenvironment, may then follow multiple pathways that culminate in cell death [21]. 

The patients’ outcome to standard radiation treatment is determined by the 5R´s of radiobiology: Repair, redistribution, repopulation, reoxygenation and radiosensitivity [22]. Repair of sublethal DNA damage is more effective in normal than tumor cells, as observed from cell recovery after exposure to ionizing radiation (IR) [22]. Radiation randomly interacts with several cellular molecules, with DNA being the main target for the biological effects of radiation, including cell killing, carcinogenesis and mutation [21]. Overall, the main goal of RT is clonogenic capacity loss [22]. Thus, radiation causes a wide range of lesions in DNA and double-strand breaks (DSB) which represents the most deleterious lesion [23]. DSBs repair might be accomplished by several mechanisms, emphasizing the importance and difficulty of repairing this type of DNA injury. 

Redistribution refers to different cellular radiosensitivity depending on cell cycle phase: S phase cells are more radioresistant than cells in late G2 and M phases [24]. Thus, small fractionated doses allow greater damage to tumor cells, due to reassortment of cells into more sensitive stages. Repopulation by increased cell division after irradiation depends on normal tissues capability to compensate killed cell populations [25]. 

Furthermore, tumor oxygenation level is a major determinant of RT effectiveness. Due to abnormal vasculature development during tumor angiogenesis, the tumor microenvironment developed areas with a decreased pH, lack of nutrients and hypoxia [26]. As a result of prolonged exposure to hypoxia, cells may acquire apoptosis resistance, which also favors clonal selection towards a more aggressive phenotype [27]. Indeed, during reoxygenation, surviving hypoxic tumor cells may increase the oxygen supply and become more radiosensitive [22]. 

During RT treatment, fractionation spares normal tissue, due to sublethal damage repair and cell repopulation occurring between treatment fractions [21]. Concomitantly, it allows increased tumor damage as a result of reoxygenation, as well as redistribution into more radiosensitive stages of the cell cycle [19]. Therefore, while DNA repair and cell repopulation induce normal tissue resistance to RT [21,28], redistribution and reoxygenation have the opposite effect increasing the radiosensitivity of tumor cells [19,22,28].

### Tumor-Associated Microenvironment: Hypoxia, HIF-1α, and Radioresistance

**Figure 1:**
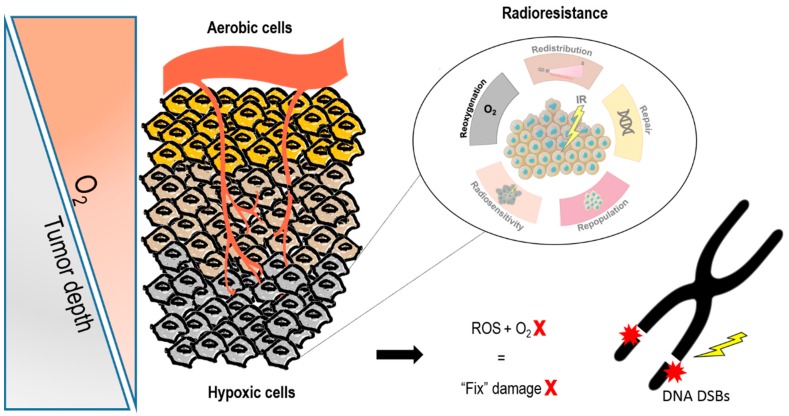
Oxygen effect on radiotherapy response. During tumor development and progression, abnormal vasculature provides a hypoxic microenvironment. Conventional radiation therapy interacts by an indirect effect, due to water radiolysis’ free radicals. Tumor reoxygenation constitutes a major determinant of radiotherapy (RT) effectiveness. Indeed, the oxygen present in the cell is a potent radiosensitizer once it participates in these free radical reactions. Thus, in the absence of oxygen, ionizing radiation effectiveness is compromised, contributing to radioresistance. Abbreviations: DSB, double strand breaks; ROS, reactive oxygen species.

Uncontrolled cell proliferation leads to oxygen demand exceeding oxygen supply. Tumor growth surpasses the capacity of the existing vasculature to provide nutrients and oxygen, leading to hypoxia. The permanent changes in blood flow and oxygen availability resulting from the tumor abnormal vasculature associate with poor prognosis and therapy resistance. In particular, RT that kills tumor cells by generating reactive oxygen species (ROS), has the ability to chemically modify radiation-induced DSB leading to permanent damage fixing [29] (Figure 1). 

Briefly, hypoxia is mediated by key transcription factors, including the hypoxia-inducible factors —HIFs that promote oxygen delivery or adaptation to oxygen deprivation, regulating the expression of over 100 genes involved in processes, such as the promotion of angiogenesis, cell proliferation, metastasis and metabolic adaptation [30]. Ubiquitously expressed oxygen-labile α subunit, HIF-1α, is known as the main hypoxia-inducible gene expression regulator [27].

Under normoxic conditions, HIF-1α is rapidly hydroxylated by prolyl-hydroxylases (PHDs), which are oxygen sensor proteins [31]. Then, these hydroxylated molecules are ubiquitinated by Von Hippel-Lindau protein (pVHL) containing E3 ubiquitin ligase, leading to HIF-1α degradation in the proteasome complex [31]. Conversely, in hypoxic conditions, HIF-1α becomes constitutively expressed owing to PHDs inhibition [31].

In chronic hypoxia, HIF-1α, through hypoxia-responsive elements (HRE), binds to functional promoter regions of several genes inducing transcriptional activation. Hypoxia is, therefore, associated with key cancer hallmarks, namely, angiogenesis, metastasis and invasion, evasion of apoptosis, metabolic reprogramming and genomic instability [26,31,32]. Overall, hypoxia and HIF-1α pathway activation are both frequently associated with therapy resistance and poor clinical outcomes [26,33]. 

In EC, high HIF-1α levels have been correlated with tumor depth of invasion, differentiation, lymphatic permeation and metastization [34]. Accordingly, HIF-1α and hypoxic-associated biomarkers, such as carbonic anhydrase IX (CAIX) and glucose transporter 1 (GLUT-1) were strongly associated with worse prognosis and poor treatment outcome of EC patient’s [13,35,36]. Furthermore, higher HIF-1α expression was reported to be associated with shorter patients’ survival and poor CRT response [33,35], and it was also extensively demonstrated that higher radiation doses are required to kill hypoxic cancer cells compared to their well-oxygenated counterparts [37]. 

Previous experiments in ESCC have suggested that HIF-1α signaling pathway’s suppression with berberine, led to tumor radiosensitization [38]. Specifically, Kato, Y et al., indicated that hypoxia leads to downregulation of homologous recombinant proteins BRCA1 and BRCA2 in EC cells, promoting G0-G1 cell cycle arrest and consequently lower radiotherapy response [39]. Indeed, cell cycle kinetics and DNA damage play a critical role in tumor radiosensitivity evaluation at hypoxia conditions. Accordingly, experimental studies are required to unveil molecular mechanisms in tumor-surrounding microenvironment that allow hypoxic cell selection, oxygenation and sensitization to allow to improve therapeutic efficacy in EC patients [33,40].

## 3. Cancer Epigenetics: A Brief Overview

Epigenetics consists of a set of heterodynamic and heritable changes which pass from cell generation to cell generation, affecting chromatin organization and transcriptional gene regulation, without altering the DNA sequence [16]. Although certain genomic mutations have an important influence on the malignant phenotype, tumor heterogeneity goes beyond somatic mutations and can often be explained by epigenetic changes. Thus, the epigenome landscape is currently recognized as having an important role in the process of neoplastic transformation and progression [41]. Furthermore, several changes in the epigenetic landscape mediate gene expression regulation and play key roles in signaling pathways which contributes to tumor development and progression [41].

**Figure 2:**
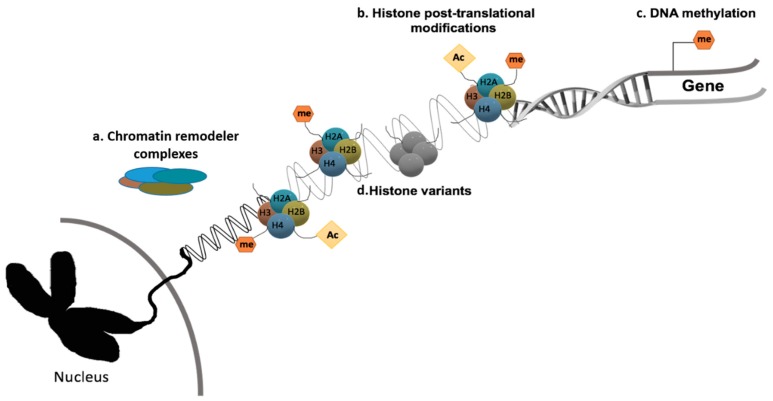
Epigenetic mechanisms. Epigenetic machinery and overall gene transcriptional deregulation. **(a)** Chromatin remodeler complexes catalyze chromatin changes. **(b)** Histone post-translational modifications are covalent dynamic changes of histones that affect chromosomal structure and function. **(c)** DNA methylation: The addition of a methyl group to CpG islands of cancer related genes’ promotor regions induce transcriptional repression. **(d)** Histone variants, proteins that replace the core canonical histone in the nucleosome, are often characteristic of specific structural and functional features in the cell.

The major epigenetic mechanisms currently known are histone post-translational modifications (PTMs), histone variants, DNA methylation and chromatin remodeling (Figure 2). The basic structure of human chromatin comprises the DNA chain coupled with histone proteins, including the histone octamer composed by H2A, H2B, H3 and H4. These specific proteins may acquire post-translational covalent alterations in C and N-terminal tails [41,42]. The most studied histone modifications are arginine’s and lysine’s methylation, acetylation and phosphorylation [41,43]. In normal cells, epigenetic mechanisms regulate constitutive gene expression, which maintains cellular variability and identity.

Several epigenetic alterations, including aberrant DNA methylation and histone onco-modifications, found in several cancers and associated with oncogene activation and tumor suppressor gene silencing [15,41], have emerged as potential cancer biomarkers [44]. Concerning PTMs, histone acetylation, which is often associated with transcriptional activation, is regulated by histone acetyltransferases (HATs) and histone deacetylases (HDAC), subdivided into four classes [45,46]. Histone methylation has a more pleiotropic effect, depending on the residue involved: Whereas, H3K4me3 and H3K36me2/3 are mostly associated with transcriptional activation, H3K9me3 and H3K27me3 are frequently associated with transcriptional repression [45,47]. Remarkably, histone methylation is a frequent event in cancer, and its dynamic transition plays a critical role in transcriptional regulation and subsequently in signaling pathways related with cell cycle, tumor metabolism and overall tumor aggressiveness capacities [48]. Accordingly, lysine methyltransferases (KMTs) and histone lysine demethylases (KDMs) regulate the methylation status of histones, and also protein substracts. KMTs, which added methyl groups in histones, comprise two major classes—those with or without SET [Su(var)3-9, E(z) (enhancer of zeste) and trithorax] domain [49]. Conversely, these methyl groups are specifically removed by several KDMs, which also comprise two major subgroups—lysine-specific demethylase (LSD1)/KDM1A containing a FAD (flavin adenine dinucleotide) dependent domain and the 2-oxoglutarate-dependent dioxygenase superfamily which encompasses most enzymes, composed by 7 KDMs subfamilies (KDM2-KDM8), with a Jumonji C catalytic domain with O_2_, Fe (II) and α-ketoglutarate as cofactor to catalyze the demethylation process [49,50]. 

Importantly, aberrant histone PTMs represents an essential contribution to EC progression and development. Briefly, HDAC6 overexpression induced higher proliferation and migration in ESCC cancer cell lines [51]. Accordingly, higher HDAC1 expression in ESCC patients with advanced stages, associated with aggressiveness [52]. Also, a significant H4 hyperacetylation in the early stage ESCC tissues was also observed [52]. However, when tumors progress and are invasive, global hypoacetylation might occur, indicating a dynamic interaction and equilibrium between HATs and HDACs in the tumor cells [52]. Particularly, in EC, several research teams suggest that histone methylation also plays a critical role in the maintenance of cells balance state, depending on the lysine residue involved. Moreover, high KMTs expression, such as SMYD3/KMT3E promotes ESCC cell proliferation. This KMT enzyme negatively interacts with RIZ1 (retinoblastoma protein-interacting zinc finger gene 1), leading to decreased H3K9 methylation levels [53]. Concerning histone demethylases, Jumonji C domain KDMs members of family GASC1/KDM4C and JMJD1C/KDM3C demethylating agents of repressive histone marker H3K9 and KDM7B/PHF8 which specifically demethylate H3K27me1/2, play a critical role in ESCC cancer development [54,55,56]. Moreover, LDS1 also contributes to EC malignant progression, leading to a patient’s poor prognosis [57,58]. Overall, high expression levels of these enzymes induce changes in several pathways, including, proliferation, migration and invasion. 

Another extensively studied epigenetic alteration occurring in cancer cells is aberrant DNA methylation [59]. This process mostly interferes with gene promoter regions to induce their downregulation [59]. Briefly, DNA methylation consists in the addition of methyl groups (-CH3) at the 5’ position of the cytosines of the DNA chain, mainly at so-called CpG islands, which are regions enriched with cytosine and guanine nucleotides [59]. Similarly, to PTMs, DNA methylation is also mediated by DNA methyltransferases (DNMTs) [59]. Particularly, DNMT1, DNMT3a and DNMT3b are the main enzymes implicated in cytosines methylation during DNA replication [59]. Importantly, DNA hypermethylation is associated with onco-suppressor genes silencing [59], whereas, promoter hypomethylation leads to oncogene overexpression and global genomic instability [59]. Specifically, *CDKN2A*, *MGMT*, *APC*, *RUNX3*, *TIMP-3* and *CRBP*1 hypermethylation have been frequently reported in EC. Aberrant DNA methylation is the most studied epigenetic mechanism in EC [60,61]. Epigenetic modifications, primarily in the form of DNA hypermethylation of cancer-related genes frequently occur in both ESCC and EAC, as well as in BE (the EAC precursor lesion), with some of these methylated tumor suppressor genes preceding the progression of BE to EAC or dysplasia to ESCC, thus, being good cancer biomarker candidates’ [61].

### 3.1. Epigenetic Remodeling and Hypoxic Microenvironment

**Figure 3:**
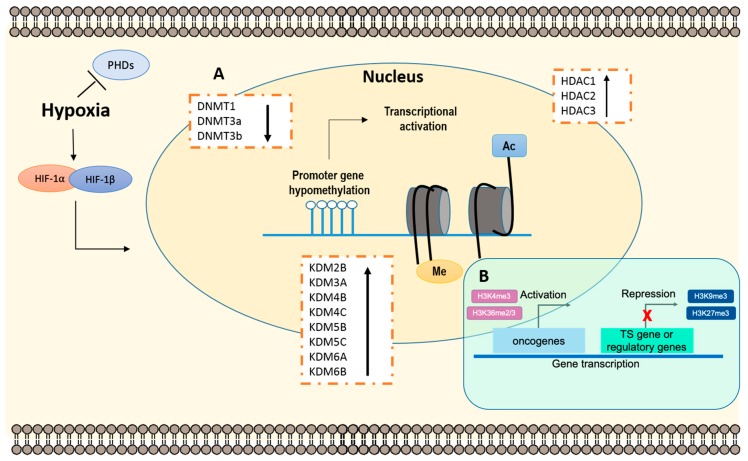
Epigenetic remodeling through hypoxic-dependent pathways. The accumulation of epigenetic alterations is characteristic of a hypoxic microenvironment in tumors. **(A)** Epigenetic changes. Briefly, is has been intensively reported that after HIF-1α stabilization, decreased DNA methyltransferases (DNMTs) activity correlates with oncogene activation. Conversely, HCADs and KDMs increased activity induces overall genomic instability. **(B)** Histone modifications. Histone methylation, depending on the residue, induces gene transcription activation or repression. Specifically, H3K4me3 and H3K36me2/3 are often correlated with transcriptional activation, whereas H3K9me3 and H3k27me3 are associated with transcriptional repression, both implicated tumor development and progression. Abbreviations: Ac—acetylation; DNMTs—DNA methyltransferases; HDACs—Histone deacetylases; HIF—Hypoxia-inducible factors; KDMs—Histone lysine demethylases; Me—methylation; PHD—Prolyl hydroxylases; TS—Tumor suppressor.

Considering tumor heterogeneity and microenvironment, hypoxia was suggested to play a critical role in epigenetic regulation. Consequently, this interaction fosters tumor progression and increased aggressiveness [62]. Indeed, activation of HIF-1α induces several cellular adaptations through oxygen-dependent manner [62]. Remarkably, hypoxia-induced aberrant DNA methylation, altered histone PTMs patterns and modifications in their respective chromatin remodelers have been reported in several neoplasms [62] (Figure 3). Furthermore, HIF-1α plays a critical role in expression regulation, through HRE binding sites at promoter regions of the genes encoding for those enzymes. Interestingly, Skowronski and co-workers demonstrated that DNMT1 and DNMT3a proteins were significantly downregulated under hypoxia, in human colorectal cancer cell lines, entailing oncogene transcriptional activation and overall genetic instability [63,64]. 

Conversely, ten-eleven translocation (TETs) dioxygenases proteins family, which mediate DNA methylation, particularly TET1 and TET3, have been reported to be overexpressed in tumor cells in association with hypoxia-induced pathways [65]. TET1 was shown to be a transcriptional co-activator associated with HIF-1α enhancement regulation in several human cancer cell lines from kidney, lung, liver and head and neck [66]. Moreover, TET proteins’ overexpression was closely associated with tumor angiogenesis, invasion and migration, since it allows for transcriptional activation of epithelial mesenchymal transition (EMT) promoting genes [66]. 

In addition, PTMs specifically associated with aggressive malignant phenotypes have been correlated with hypoxia and HIF-1α activation [62]. Accordingly, hypoxia was implicated in decreased histone acetylation levels, which are involved in overall gene transcriptional repression [67]. In accordance, global HDACs activity is modulated in hypoxic condition [67]. For instance, HDAC1 was associated with HIF-1α signaling pathways, being significantly overexpressed in the Lewis lung carcinoma model, increasing angiogenesis [67]. In accordance, Kim et al. have demonstrated the critical role of HDACs-induced hypoxia in angiogenesis and tumor progression [67]. Indeed, HDAC1, HDAC2 and HDAC3 were upregulated under hypoxic microenvironment in human kidney and lung carcinoma cell lines [67], and thus, HDAC inhibitors (HDACi) were suggested as a potential therapeutic agent in tumors with significant hypoxic regions [67].

Furthermore, several studies have identified genome binding sites for other key regulators, namely, JmjC-KDMs, and some of them were proposed as novel therapeutic targets in several cancer models [68]. Hypoxia-induced KDM3A, KDM4B, KDM4C (specifically in EC [54]), KDM6A, KDM6B, KDM2B, KDM5B and KDM5C overexpression [69,70]. Accordingly, Ikeda et al. demonstrated that KDM3A/JMJD1A was tightly regulated by HIF-1α, under hypoxic conditions, in multiple myeloma cell lines [71]. These dynamic alterations led to anti-apoptotic phenomena, invasive and pro-angiogenic properties [72,73]. Moreover, the KDM4 subfamily, particularly KDM4C/JMJD2C, which target methylated H3K9 and H3K36 histone marks, contributes to cancer initiation and development, and is associated with hypoxic status in breast cancer [74,75], whereas, in renal cell carcinoma cell lines, KDM3A, KDM4B and KDM5B were shown to be regulated by HIF family. Also, KDM6B/JMJD3, which demethylate H3K27me3, was previously demonstrated to play a critical role in the hypoxic signaling pathway, in the human liver and kidney cell lines [76,77]. The involvement of these main JmjC-KDMs highlight the importance of HIF-1α-dependent epigenetic regulation in hypoxia, leading to several molecular changes, like cell cycle arrest and DNA repair, which contribute to cancer progression. 

In EC, the interplay between the hypoxia and epigenetic mechanism regulation remains poorly understood. Considering that both hypoxia and epigenetic alterations are common features in EC, however, its relevance in this particular cancer type is very likely [35]. HDAC1/2 were previously found to be deregulated in EC, and HDAC activity was associated with in vitro anti-neoplastic activity, in human EC cell lines [78]. Conversely, KDM4C/JMJD2C/GASC1 overexpression induced EC initiation and development [54]. Although this epigenetic mechanism is intensively studied in EC, the association of these alterations with tumor hypoxic microenvironment has not been reported, yet. Nonetheless, KDM3A/JMJD1A and KDM4C/JMJD2C were suggested to be hypoxia-inducible genes in kidney and prostate cancer cell lines [79]. Although it is acknowledged that hypoxia confers resistance to RT and that some epigenetic alterations are regulated by HIF-1α, the interaction between the hypoxia and epigenetic mechanism remains poorly understood, particularly in EC.

### 3.2. Epigenetic Modifications and Radiosensitivity

**Figure 4:**
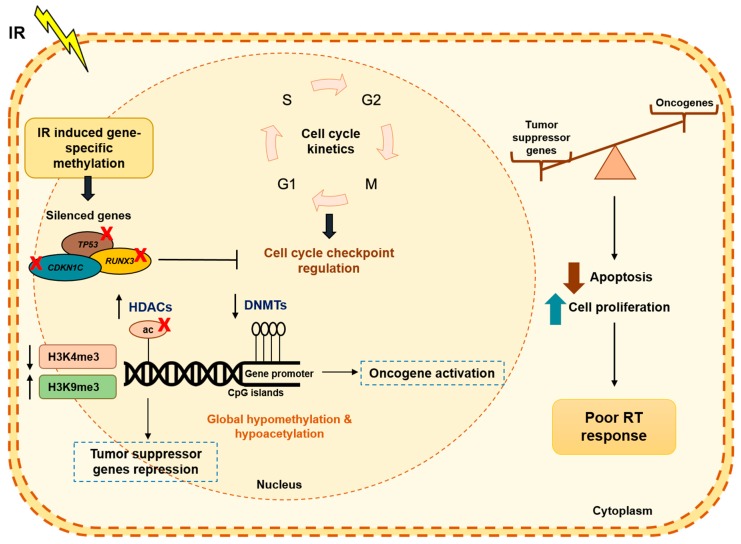
Radiation-induced epigenetic alterations. A phenotypic switch during radiation treatment, leads several epigenetic modifications in the tumor cells. In fact, DNA methylation has been the most studied mechanism in RT resistance. Changes in DNA methylation did not occur uniformly into the cell genome. It has been described IR-induced specific methylated genes as *TP53, CDKN1C* and *RUNX3,* among others, in EC, resulting in uncontrolled cell cycle checkpoints and tumor growth. Additionally, PTMs, such as histone acetylation and/or methylation are also deregulated after IR treatment to promote cancer progression and development. Accordingly, there is a dynamic balance between tumor suppressor genes and oncogenes, resulting in lower apoptosis and higher cell proliferation and consequently, poor RT response. Abbreviations: Ac—acetylation; *CDKN1C*—cyclin dependent kinase inhibitor 1C; DNMTs—DNA methyltransferases; HDAC—histone deacetylases; IR—ionizing radiation; RT—radiotherapy; *RUNX3*—runt related transcription factor 3; *TP53*—tumor protein p53.

The role of epigenetic modifications, especially DNA methylation, has been widely implicated in radiation resistance, playing a critical role in cell cycle progression and checkpoint regulation, DNA repair and apoptosis, all of which decisive in determining radiation cell response (Figure 4). Recently, dysregulated epigenetic control through DNA methylation was associated with tumors’ radiation resistance [80]. Exposure to IR results in DNMTs decreased levels. 

Indeed, decreased global DNA methylation was reported in tumor cells after IR. Moreover, in the course of DNA repair induced IR, polymerases are not able to incorporate methylcytosine during DNA synthesis, but rather cytosine [81]. Thus, a global DNA hypomethylation has been observed after IR and was associated with oncogene transcription activation and consequently carcinogenesis [81].

In EC, increased methylation levels of specific genes, such as *TP73* (tumor protein p53), *CDKN1C* (cyclin dependent kinase inhibitor 1C) and *RUNX3* (runt related transcription factor 3), among others, were significantly correlated with poor radiation response, resulting in uncontrolled cell growth [82]. *RUNX3*, a tumor suppressor mediating TGF-β-dependent apoptosis pathway was reported to be hypermethylated and downregulated in radioresistant EC cells. Thus, the assessment of respective expression and methylation levels in pretreatment samples might be useful for predicting ESCC radiosensitivity [83].

Furthermore, some histone PTMs may also affect cellular response to external IR stimuli. Indeed, global histone hypoacetylation, through increased HDAC activity and decreased H3K4me3 transcriptional activation-associated marker, were already associated with radioresistance [84,85]. Conversely, increased H3K9me3, a transcriptional repressive-associated marker [85], paralleling DNA methylation studies, was also associated with therapy resistance and tumor progression. 

Despite increasing evidence of an association between epigenetic changes and radiosensitivity, there are no epigenetic biomarkers used in clinical practice for determining RT response. Some epigenetic drugs that might be used as radiosensitizers (such as DNMTi or HDACi, among others), are still being evaluated, although recent studies suggest their increased efficacy under hypoxic conditions.

## 4. Emerging Novel Therapeutic Targets through Epigenetic Chromatin Modulation under Hypoxia

Interestingly, the development of new therapeutic strategies targeting epigenetic effectors is progressively contributing to the discovery of novel molecular biomarkers [86]. Although several epigenetic targeting drugs, which reverse aberrant DNA methylation and histone onco-modifications, have been approved for clinical use [86] (Table 1), new epigenetic-based biomarkers, predictive of response to RT, and consequently, of radiosensitization of hypoxic cells, remain mostly unexplored, particularly in EC.

DNA methylation inhibition, using DNMTi, has been emerged as a potential therapeutic strategy in combination with radio- and chemotherapy [87]. Indeed, after IR exposure, significant alterations in DNA methylation status occur [87]. Thus, well established DNMTi, like Azacytidine (AZA) and Decitabine (DAC), might improve therapy response in several cancers through transcriptional reactivation of tumor suppressor genes [88]. Currently, those drugs, which were approved by Food and Drug Administration (FDA) and European medicine agency (EMA) for treatment of myelodysplastic syndrome (MDS) and acute myeloid leukemia (AML) [86], are being tested in EC. Indeed, Ahrens et al. showed, in vitro, that combination of AZA with HDACi, such as SAHA, MS-275 and FK228, leads to a significant decrease in tumor cell viability [78] (Table 1). Accordingly, clinical trials testing the combination of DNMTi and HDACi to improve chemoradiation patient’s outcome are currently ongoing. With this purpose, Hydralazine plus Valproate or Valproic acid (VPA), an anti-epileptic drug, were used in clinical trials phase II for cervical cancer (NCT00404326) [89]. Herein, patients obtained a complete response to external radiation, thus, supporting further investigation [89]. Moreover, HDAC class I and II inhibitors, such as Vorinostat and VPA in combination with RT showed to increased tumor radiosensitization [90,91]. In particular, a phase I clinical trial with Panobinostat, an epidrug that function as HDAC inhibitor, sensitized prostate, head and neck and Esophageal tumors for external RT (NCT00670553) [90] (Table 1). 

Additionally, Jonsson et al. showed that HDACi treatment-induced radioresistant-associated genes deregulation, including HIF-1α downregulation, in in vitro studies [90]. Also, Trichostatin A (TSA) treatment was found to decrease HIF-1α levels in tumor cells [92]. Nevertheless, the molecular mechanism subjacent to hypoxia and HDACs activity is still lacking [90,93].

Likewise, histone methylation remodelers upregulated during hypoxia stabilization, were also suggested as targets for cancer therapy [94]. Specifically, hypoxia-induced JmjC-KDMs, which are HIF-1α direct targets, were pinpointed as potential candidates for therapeutic intervention [62,94]. Glioblastoma, characterized by extensive hypoxia were reported to display KDM5 subfamily upregulation, which specific inhibition induced cell cycle arrest and apoptosis associated with improved overall survival rates [95]. KDM4 family members are attractive therapeutic targets for EC, because KDM4C has been strongly implicated in tumor initiation and progression [96]. Remarkably, KDM4 inhibitors, such as carboxylic acid inhibitors, including GSK, compound 3–6 and 2,4-PDCA, demonstrated substantial antiproliferative effects in EC cell lines [96] (Table 1). Therefore, advances in the development of new KDMs inhibitors have been reported to improve cancer survival [97]. In particular, inhibitors targeting demethylating remodelers belonging to LSD1 subgroup, including KDM1A, are currently tested in Phase I clinical trials [97]. In accordance, tranylcypromine (TCP), structurally similar to chemicals previously used in the treatment of other disorders, including Parkinson’s disease, has been tested as KDM1 inhibitor [97] (Table 1). Furthermore, a broad-spectrum of pan KDM inhibitors, most of them 2OG mimic, like NOG, SAHA, IOX1, 2,4-PDCA and JIB-04, have demonstrated significant anti-neoplastic potential [97]. JIB-04 was shown to radiosensitize lung squamous cell carcinoma cell lines by decreasing DNA repair activity after IR therapy [98]. Moreover, specific inhibitory drugs have been developed and are currently in pre-clinical trials, including EPT103182, a KDM5 inhibitor in hematological cancers, such as multiple myeloma, and GSK-J1, a KDM6 inhibitor, in glioma, both with high efficacy against cancer cells [97,99] (Table 1). RS compounds were suggested to increase breast cancer cells sensitivity to RT, affecting DNA repair mechanisms [100] (Table 1). Additionally, KDM6 subfamily inhibitors, such as GSK-J1/J4, also enhanced glioblastoma cells radiosensitivity, as well as, in breast and lung adenocarcinoma cell lines [99] (Table 1). 

Overall, according to the premise that epigenetic modulation promotes radiation resistance under hypoxic microenvironment, additional pre-clinical and clinical trials are required to confirm the efficacy and safety of new epigenetic drugs that may increase cancer cells’ radiosensitivity, particularly in hypoxic microenvironment. 

## 5. Conclusions

For many years, research in Oncology has been focused on the identification of key targetable molecules among the most relevant signaling pathways, which might allow for the implementation of Precision Medicine. Because RT is the standard of care for treatment of many cancer types, including EC, new complementary therapeutic strategies are needed to overcome radioresistance. Indeed, hypoxic microenvironment, mainly driven by HIF-1α activation, drastically reduces cell radiosensitivity. Furthermore, epigenetic mechanisms involved in the dynamic regulation of chromatin induce a hypoxic microenvironment, triggering a series of cellular processes, including cell cycle alterations, increased migration and invasion, angiogenesis, DNA repair adaptations and cell survival. Therefore, the discovery of new molecular epigenetic targets implicated in hypoxia-responsive pathways seems to be key for improving the management and survival of EC patients.

## Figures and Tables

**Table 1:** Epigenetic targeting drugs for clinical use. Current link between hypoxic-dependent modulation and radiotherapeutic improvement.

Epigenetic Drug	Target Function	Role in EC Treatment (Yes/No)	RT Response Improvement (Yes/No)	Study with Hypoxia Linked (Yes/No)	Other Models	References
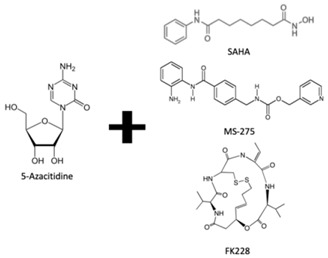	DNMTi combined with HDACi	Yes	Yes	No	-	[78]
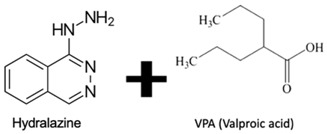	DNMTi combined with HDACi	No	Yes	No	Cervical cancer	[89]
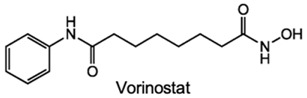	HDACi	No	Yes	Yes	Prostate cancer	[90]
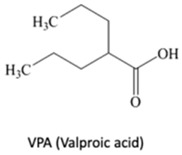	HDACi	Yes	Yes	No	-	[91]
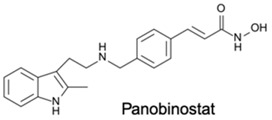	HDACi	Yes	Yes	No	Prostate cancer and head and neck tumors	[90]
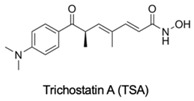	HDACi	No	No	Yes	-	[92]
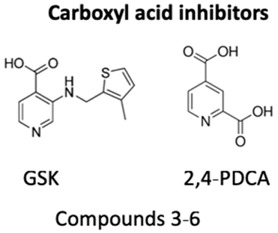	KDM4 inhibitors	Yes	No	No	-	[96]
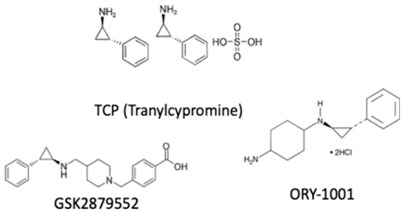	LSD inhibitors	No	No	No	Acute myeloid leukemia (AML) and small cell lung carcinoma (SCLC)	[97]
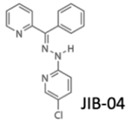	KDM5B inhibition (JmjC-KDM PAN inhibitor)	No	Yes	No	Lung cancer	[98]
EPT103182	KDM5 subfamily inhibitor	No	No	No	Multiple myeloma	[97]
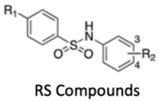	KDM5 subfamily inhibitors	No	Yes	No	Breast cancer	[100]
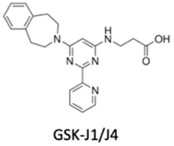	KDM6 subfamily inhibitor	No	Yes	No	Glioblastoma, breast and lung adenocarcinoma	[99]

^1^ Abbreviations: AML—Acute myeloid leukemia; DNMTi—DNA methyltransferase inhibitor; EC—Esophageal cancer; GSK—Glycogen synthase; HDACi—Histone deacetylase inhibitor; JmjC-KDM—Jumonji contain c domain histone lysine demethylase; KDM—Histone lysine demethylase; kinase; LSD—Lysine-specific demethylase; PDCA—Pyridinedicarboxylic acid; RT—radiotherapy; SAHA – suberoylanilide hydroxamic acid; TCP—tranylcypromine; TSA—Trichostatin A; VPA—Valproic acid. table
